# The advantages of the magnetic resonance image compilation (MAGiC) method for the prognosis of neonatal hypoglycemic encephalopathy

**DOI:** 10.3389/fnins.2023.1179535

**Published:** 2023-06-15

**Authors:** Zhongfu Tian, Qing Zhu, Ruizhu Wang, Yanli Xi, Wenwei Tang, Ming Yang

**Affiliations:** ^1^Department of Radiology, Women's Hospital of Nanjing Medical University (Nanjing Maternity and Child Health Care Hospital), Nanjing, China; ^2^Department of Radiology, Children’s Hospital of Nanjing Medical University, Nanjing, China

**Keywords:** synthetic MRI, magnetic resonance image compilation (MAGiC), hypoglycemic encephalopathy, neonatal, prognosis

## Abstract

**Objectives:**

To explore the prognostic value of magnetic resonance image compilation (MAGiC) in the quantitative assessment of neonatal hypoglycemic encephalopathy (HE).

**Methods:**

A total of 75 neonatal HE patients who underwent synthetic MRI were included in this retrospective study. Perinatal clinical data were collected. T1, T2 and proton density (PD) values were measured in the white matter of the frontal lobe, parietal lobe, temporal lobe and occipital lobe, centrum semiovale, periventricular white matter, thalamus, lenticular nucleus, caudate nucleus, corpus callosum and cerebellum, which were generated by MAGiC. The patients were divided into two groups (group A: normal and mild developmental disability; group B: severe developmental disability) according to the score of Bayley Scales of Infant Development (Bayley III) at 9–12 months of age. Student’s *t* test, Wilcoxon test, and Fisher’s test were performed to compare data across the two groups. Multivariate logistic regression was used to identify the predictors of poor prognosis, and receiver operating characteristic (ROC) curves were created to evaluate the diagnostic accuracy.

**Results:**

T1 and T2 values of the parietal lobe, occipital lobe, center semiovale, periventricular white matter, thalamus, and corpus callosum were higher in group B than in group A (*p* < 0.05). PD values of the occipital lobe, center semiovale, thalamus, and corpus callosum were higher in group B than in group A (*p* < 0.05). Multivariate logistic regression analysis showed that the duration of hypoglycemia, neonatal behavioral neurological assessment (NBNA) scores, T1 and T2 values of the occipital lobe, and T1 values of the corpus callosum and thalamus were independent predictors of severe HE (OR > 1, *p* < 0.05). The T2 values of the occipital lobe showed the best diagnostic performance, with an AUC value of 0.844, sensitivity of 83.02%, and specificity of 88.16%. Furthermore, the combination of MAGiC quantitative values and perinatal clinical features can improve the AUC (AUC = 0.923) compared with the use of MAGiC or perinatal clinical features alone.

**Conclusion:**

The quantitative values of MAGiC can predict the prognosis of HE early, and the prediction efficiency is further optimized after being combined with clinical features.

## Introduction

Neonatal hypoglycemic encephalopathy (HE) is brain damage caused by decreased blood glucose concentration and insufficient energy supply in newborns and is a common metabolic disease with a lack of specificity in early clinical manifestations (Kapoor et al., 2020). Intractable and persistent hypoglycemia can cause permanent damage to children’s central nervous system, such as developmental disability, cerebral palsy, cognitive impairment, microcephaly, visual impairment, and intractable epilepsy, which seriously affects children’s health (Gu et al., 2019). Magnetic resonance imaging (MRI) has high sensitivity and is of great value in the diagnosis of HE. Functional MR imaging, such as diffusion weighted imaging (DWI) and magnetic resonance spectroscopy (MRS), can provide quantitative information to describe the complicated physiologic state of the tissue. DWI reflects the diffusion of water in neurons and glial cells according to the apparent diffusion coefficient (ADC). It plays an important role in the evaluation of neonatal HE and prognostic follow-up (Zhang et al., 2022) and can be used for quantitative evaluation of neonatal HE (Landais, 2015). MRS showed that the inverted Lac peak increased significantly and NAA/Cr decreased significantly in the occipital lobe. These metabolic changes may be related to the pathological mechanism of irreversible damage to the occipital lobe caused by repeated severe hypoglycemia (Parmentier et al., 2022). MRS is of great significance for the early understanding of the functional state of neurons (Behar et al., 1985; Azzopardi and Edwards, 1995); however, MRS takes a long time to scan and is not widely used in HE.

Synthetic MR (syMRI) is a technique that synthesizes contrast-weighted images from multicontrast MRI data. The technology has been developing rapidly since the technique was introduced. Magnetic resonance image compilation (MAGiC), provided by General electric health care (GEHC, USA), is one form of syMRI. On the basis of the 2D fast spin echo technique, MAGiC adopts the multidynamic and multiecho (MDME) principle and uses alternating 120° saturated pulse degrees and multiecho collection. Five quantitative maps and 10 contrast maps can be obtained simultaneously by one scan, thereby quantitatively measuring the relaxometry metric values (T1, T2 and proton density [PD] values), reflecting the absolute quantification of physical properties of tissue and markedly decreasing the scan time (Hwang and Fujita, 2022; Jaen-Lorites et al., 2022; Zhang et al., 2022). In contrast to functional MRI techniques, which can be affected by the scanning parameters and the biophysical model for data postprocessing, the quantitative syMRI technique is inherent and independent of MRI methods and parameters (Hagiwara et al., 2019; Ji et al., 2022).

Initial research on syMRI reported that the quality of its diagnostic images is comparable to conventional multicontrast images in the brain (Tanenbaum et al., 2017; Di Giuliano et al., 2020). In pediatric imaging, quantitative information may enable us to recognize developmental trajectories or identify pathological changes in the brain, to assess the severity of disease, to predict the prognosis and to monitor the treatment process objectively (Lee et al., 2018; Andica et al., 2019). MAGiC brain tissue volumetry has also shown favorable repeatability with different in-plane resolutions and geometries. Based on the current literature, the feasibility of generating T1, T2, and PD maps from syMRI for quantitative analysis of neonatal HE has not yet been reported.

The MAGiC method was used for this study. We retrospectively analyzed the clinical and imaging data of 75 newborn children with neonatal HE, and the main goal was to explore the predictive value of quantitative mapping generated by MAGiC for the prognosis of neonatal HE. In addition, we compared the diagnostic efficacy of MAGiC and MAGiC combined with perinatal clinical features.

## Materials and methods

### Enrolled patients

**Figure 1:**
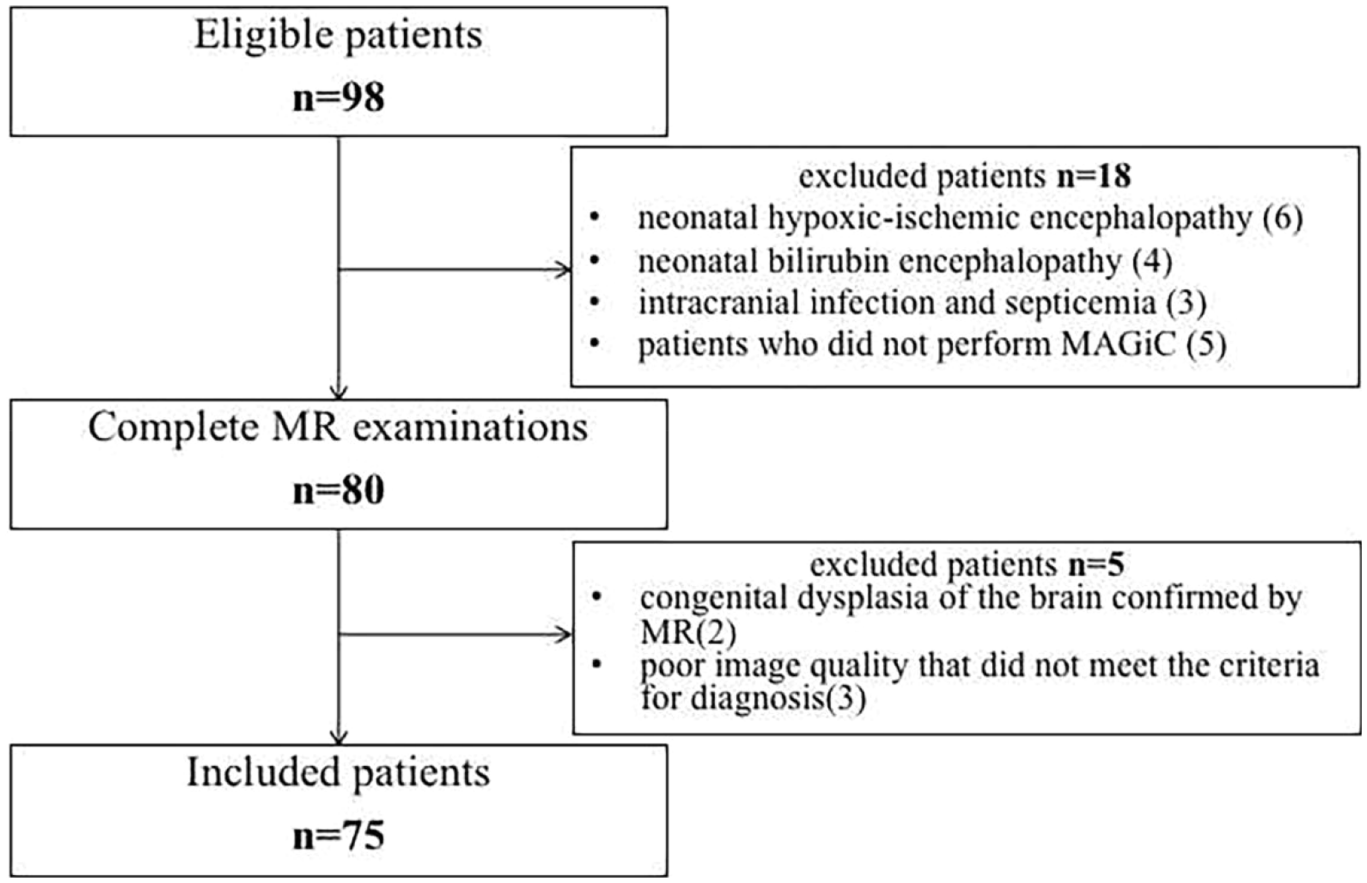
Flow diagram of enrolled patients.

The Ethics Committee of the Children’s Hospital of Nanjing Medical University/Women’s Hospital of Nanjing Medical University approved this study, and all patients’ parents provided informed consent. From July 2019 to March 2022, 75 patients diagnosed with neonatal HE in the department of neonatology were included in the study (Figure 1). The inclusion criteria were as follows: (1) venous blood glucose ≤2.2 mmol/L was recorded ≥2 times (Thompson-Branch and Havranek, 2017; Comtié de Estudios Feto-Neonatales, 2019); (2) patients had neurological symptoms, including poor reaction, vomiting, lethargy, coma, convulsion, irritability or apnea; (3) MRI or ambulate electroencephalogram showed signs of brain injury (Thompson-Branch and Havranek, 2017; Gu et al., 2019; Parmentier et al., 2022); and (4) mean neonatal gestational age ≥ 38 weeks and age ≤ 28 days at the time of admission. The exclusion criteria were as follows: (1) congenital dysplasia of the brain; (2) neonatal bilirubin encephalopathy; (3) neonatal hypoxic–ischemic encephalopathy; (4) intracranial infection and septicemia; and (5) poor image quality that did not meet the criteria for diagnosis.

Perinatal clinical data were collected, including sex, gestational age, age at MRI scan, birth weight, method of delivery (eutocia or cesarean section), history of asphyxia at birth, gestational diabetes, gestational hypertension, minimum glycemia, duration of hypoglycemia (blood glucose monitoring every 6 h), and neonatal behavioral neurological assessment (NBNA) scores. The NBNA was developed by Professor Bao Xiulan of Peking University (Bao, 2003) and includes 20 items: 6 items assessing behavioral abilities, 4 items assessing active muscle tone, 4 items assessing passive muscle tone, 3 items assessing primitive reflexes, and 3 items assessing general responses. Each item is rated on a scale ranging from 0 to 2, and the maximum score is 40 points. A score of 35 or greater is normal, and a score less than 35 is abnormal. Lower scores indicate more severe neurological issues.

At 9–12 months of follow-up, all patients were assessed by two child care physicians using the Bayley Scales of Infant Development 3rd edition (Bayley III) (Lowe et al., 2012; Sharp and DeMaruo, 2017). The patients were divided into two groups: group A was the normal group (mental and psychomotor development index ≥85) and mild developmental disability group (mental development index or psychomotor development index between 70 and 84); group B was the severe developmental disability group (mental development index or psychomotor development index ≤69).

### MRI protocol

Based on the GE Signa Architect 3.0-Tesla (T) MRI scanner, with a digital head coil, the neonates were sedated with 5% chloral hydrate 1 ml/kg before the examination and were scanned after 20–30 min when they slept. A neonatologist always escorted the patient from transport to the MRI unit to the end of imaging. MRI examination included conventional sequences and MAGiC. The parameters of the conventional sequence are as follows: three-dimensional T1-weighted image with the sagittal plane (repeat time [TR] 7–7.5 ms, echo time [TE] 3–3.5 ms); a matrix of 256 × 256, field of vision (FOV) 220 × 220 mm, layer thickness of 1 mm, and layer spacing of 1 mm. The axial plane images included T2W fast spin–echo (TR 6430 ms, TE 99 ms), T2W fluid attenuated inversion recovery (FLAIR) (TR 8,000 ms, TE 109 ms), DWI (TR 400 ms, TE 8.8 ms), gradient factors b = 0 s/mm^2^, 800 s/mm^2^, matrix 256 × 256, field of view (FOV) 220 × 220 mm, layer of thickness 4.5 mm, layer spacing of 1 mm. The MAGiC data for HE were acquired using MDME. The MAGiC/MDME is based on the quantification of relaxation times and proton density by multiecho acquisition of a saturation-recovery using Turbo spin-Echo readout (QRAPMASTER), a new sequence suggested by Warntjes et al. (2008). In QRAPMASTER, the continuous variation in slice-selective saturation and acquisition readout produces a very efficient method for measuring T1, T2, and PD (Hagiwara et al., 2019; Ji et al., 2022). A fixed TR of 4,000 ms, two TEs (effective TE 21.1 and 105.7 ms), echo chain length of 16, layer thickness of 4.5 mm, layer spacing of 1 mm, layer number of 20, FOV of 220 mm × 220 mm, matrix of 320 × 224, bandwidth of 32.15, and scanning time of 3 min 40 s; after scanning, the MAGiC was postprocessed on a syMRI 8.0 postprocessing workstation, and T1, T2 and PD values were automatically generated.

### Quantitative analysis

Quantitative maps with any combination of TE, TR and TI can be used to synthesize multiple contrast-weighted images using MAGiC, including T1, T2, T1 FLAIR, T2 FLAIR, STIR, phase-sensitive inversion recovery (PSIR) and double inversion recovery (DIR) images. Five sets of quantitative maps were generated: T1 mapping, T2 mapping, R1 mapping, R2 mapping, and PD mapping (Warntjes et al., 2008; Tanenbaum et al., 2017; Zhang et al., 2022).

Two radiologists (WRZ with 13 years and TZF with 15 years of experience in the neurology MR imaging) who were aware of perinatal clinical characteristics reviewed the images, and a consensus was reached after discussion. The final evaluation based on the average of the two radiologists was used for the final statistical analysis. The white matter of the frontal lobe, parietal lobe, temporal lobe, occipital lobe, centrum semiovale, periventricular white matter, thalamus, lentiform nucleus, caudate nucleus, corpus callosum and cerebellum were selected as the study sites, and bilateral symmetry was applied at each site, except for the corpus callosum. We selected the genu and splenium as regions of interest (ROIs) in the corpus callosum. Three ROIs were delineated evenly for each anatomical part, with a size of 10 ± 3 mm^2^, and the mean value was used for statistics. The position and size of the ROI of each patient should be as consistent as possible. In total, 66 ROIs were analyzed in this study. The mean values of T1 (ms), T2 (ms) and PD (pu) at each site were collected for statistical analysis. ROIs should be selected as far away from the brain sulci, cisterna, and cerebrospinal fluid (CSF) as possible.

### Statistical analysis

SPSS Statistics Version 22.0 was used for statistical analysis. The interobserver consistency between the two readers was assessed by the interclass correlation coefficient (ICC). It is generally considered that an ICC below 0.4 indicates poor consistency, whereas an ICC greater than 0.75 indicates good consistency. The Kolmogorov–Smirnov test was used to examine the normality of the data. The homogeneity of variance was evaluated by the Levene test. Student’s *t* test was used for comparisons between groups for which the data conformed to a normal distribution. The Wilcoxon test was employed in comparisons between groups in which the data were skewed. Categorical data were expressed as frequencies, and Fisher’s exact test was used to compare the two groups. Receiver operating characteristic (ROC) curve analysis was performed to obtain the optimum threshold value, sensitivity, and specificity. The area under the curve (AUC) was computed to evaluate the diagnostic accuracy of the quantitative parameters derived from MAGiC. Significant parameters (*p* < 0.05) were entered into a multivariate binary logistic regression model to construct a multiparameter diagnostic model of MAGiC and MAGiC + perinatal clinical characteristics. Formulas with significant parameters and coefficients were generated to predict the prognosis of neonatal HE. The DeLong test was performed to compare the AUC values. *p* < 0.05 indicated statistical significance.

## Results

### Demographic data and perinatal clinical characteristics

A total of 75 participants (aged 38.1–39.5 weeks), comprising 37 boys and 38 girls, were enrolled in this study. The demographic and perinatal clinical characteristics are shown in Table 1. There were no significant differences in sex, gestational age, age at MAGiC scanning, method of delivery, or minimum glycemia between the two groups (*p* > 0.05). There were significant differences in birth weight, history of asphyxia at birth, gestational diabetes or gestational hypertension, duration of hypoglycemia and NBNA scores between the two groups (*p* > 0.05).

**Table 1:** Demographic and perinatal clinical characteristics in the two groups.

Characteristic	Group A (*n* = 40)	Group B (*n* = 35)	*F*	*P*
Male:female	21:19	16:19	–	0.211
Gestational age (weeks, *x* ± *s*)	38.1 ± 0.3	38.5 ± 0.2	2.09	0.121
Age at MAGiC scan (days, *x* ± *s*)	5.8 ± 2.7	6.2 ± 1.5	1.23	0.354
Birth weight (g, *x* ± *s*)	3250 ± 130	3020 ± 220	−3.10	0.015
Method of delivery (cesarean section/eutocia)	29/11	26/9	–	0.091
History of asphyxia at birth (*n*)	4	8	–	0.010
Gestational diabetes /gestational hypertension (*n*)	10	19	–	0.002
Minimum glycemia (mmol/L, *x* ± *s*)	1.0 ± 0.1	1.2 ± 0.2	−2.34	0.089
Duration of hypoglycemia (d, *x* ± *s*)	5.0 ± 0. 2	9.2 ± 0.4	−3.09	0.030
NBNA score (*x* ± *s*)	30.0 ± 3. 2	22.0 ± 2. 6	−4.21	0.010

NBNA, neonatal behavioral neurological assessment; − Fisher’s exact test.

### Quantitative analysis

**Figure 2:**
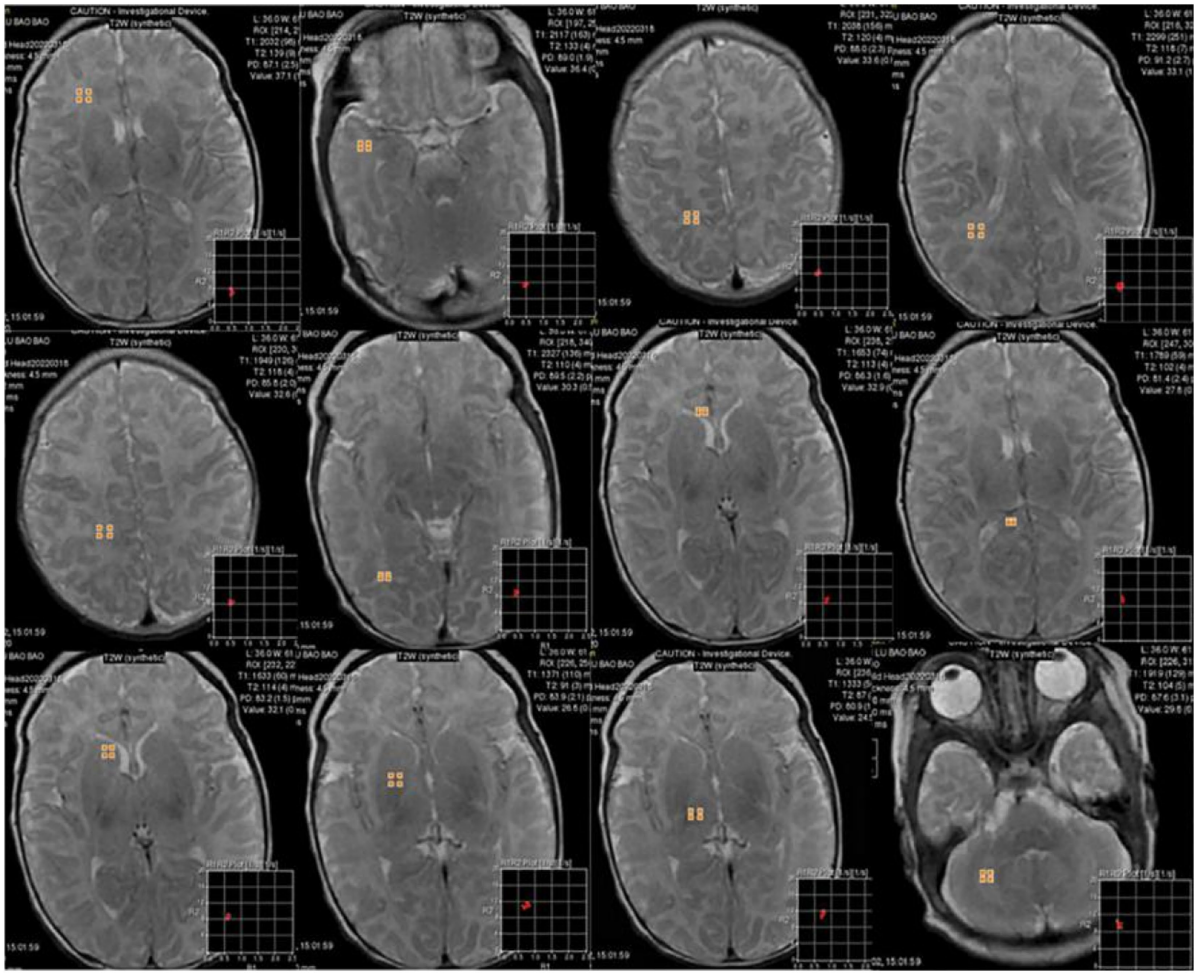
6-day-old male neonate with HE: synthetically generated T2w images using MAGiC. ROIs were delineated in the white matter of the frontal lobe, temporal lobe, parietal lobe, periventricular white matter, centrum semiovale, occipital lobe, corpus callosum, lentiform nucleus, caudate nucleus, thalamus, and cerebellum. Three ROIs were delineated for each anatomical part except corpus callosum, with a size of 10 ± 3  mm^2^, and the mean value was used for statistical analysis.

**Figure 3:**
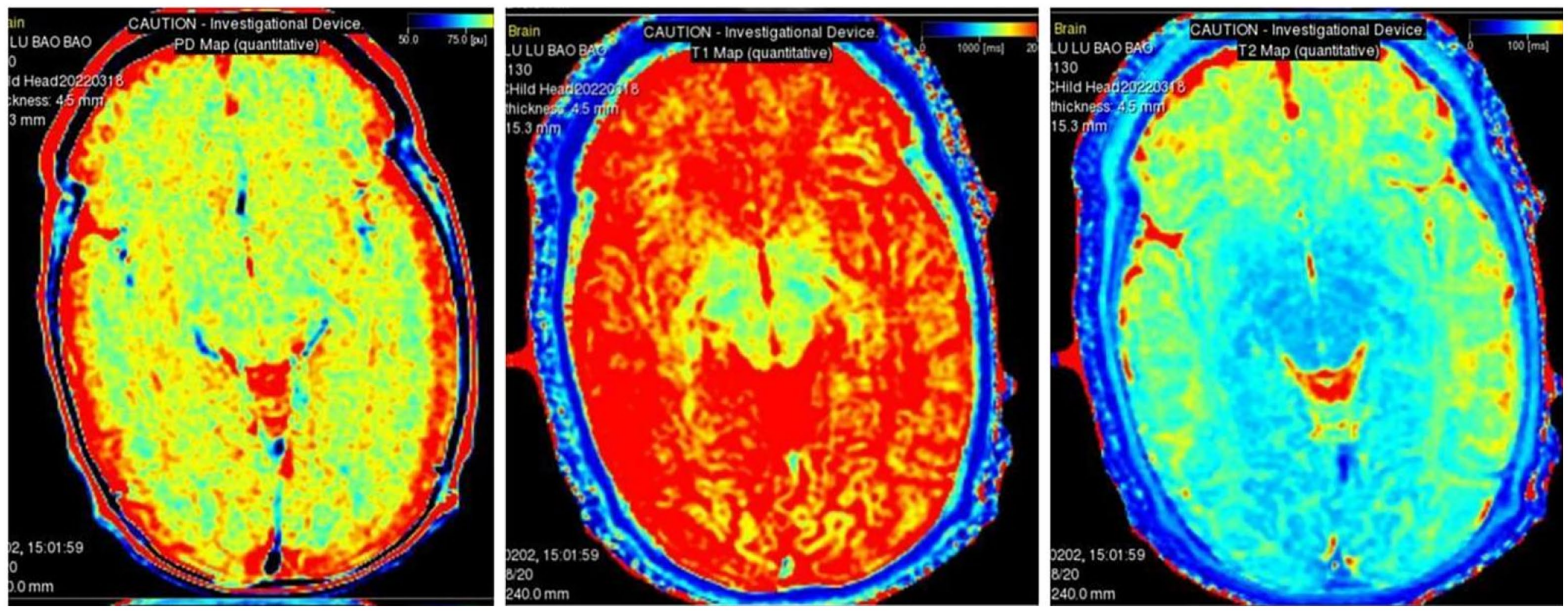
MAGiC-generated quantitative PD mapping, T1 mapping and T2 mapping.

**Figure 4:**
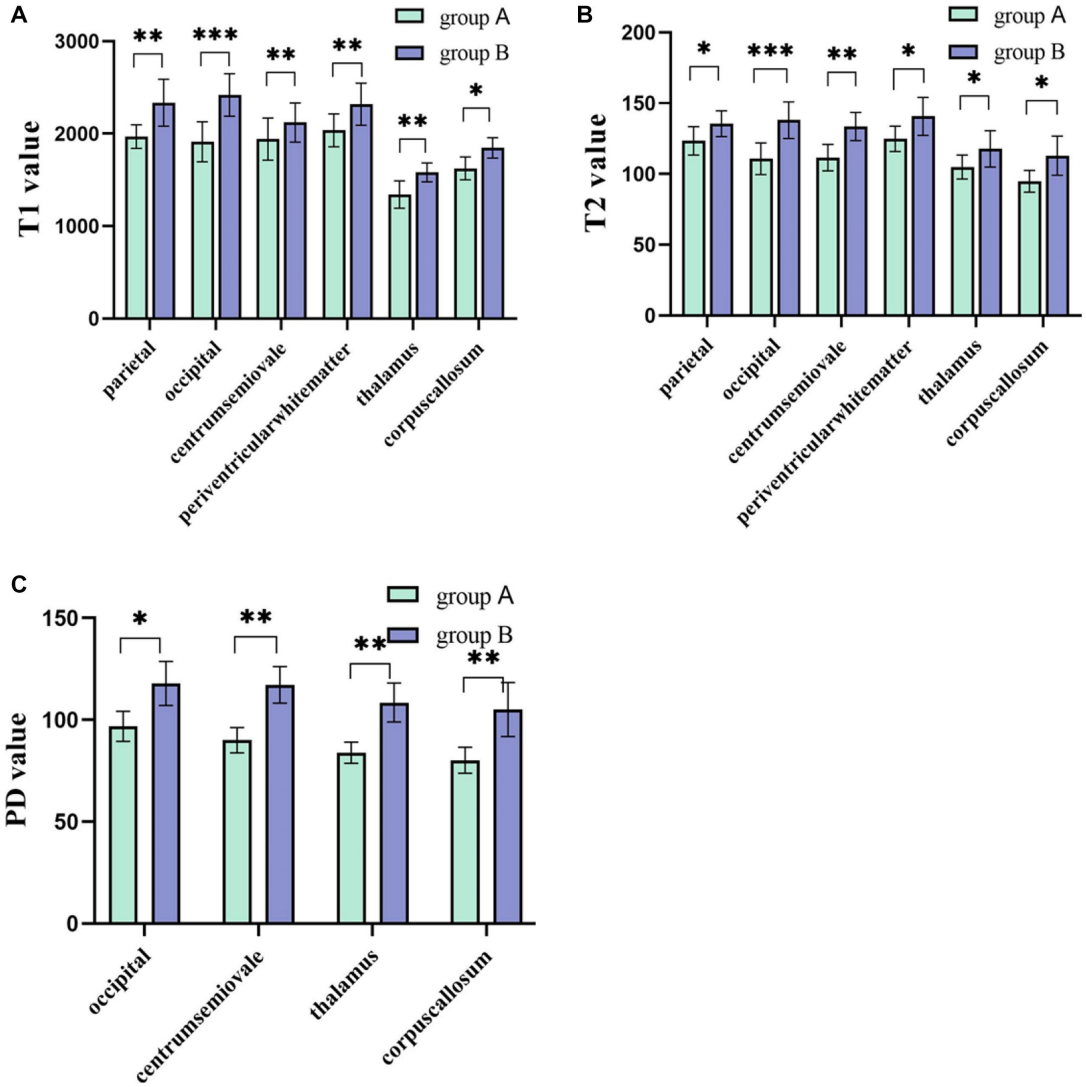
**(A,B)** The T1 and T2 values of the parietal lobe, occipital lobe, center semiovale, periventricular white matter, thalamus, and corpus callosum were higher in group B than in group A (*p* < 0.05). **(C)** The PD values of the occipital lobe, center semiovale, thalamus, and corpus callosum were higher in group B than in group A (*p* < 0.05). *p < 0.05; **p < 0.01; ***p < 0.001.

**Figure 5:**
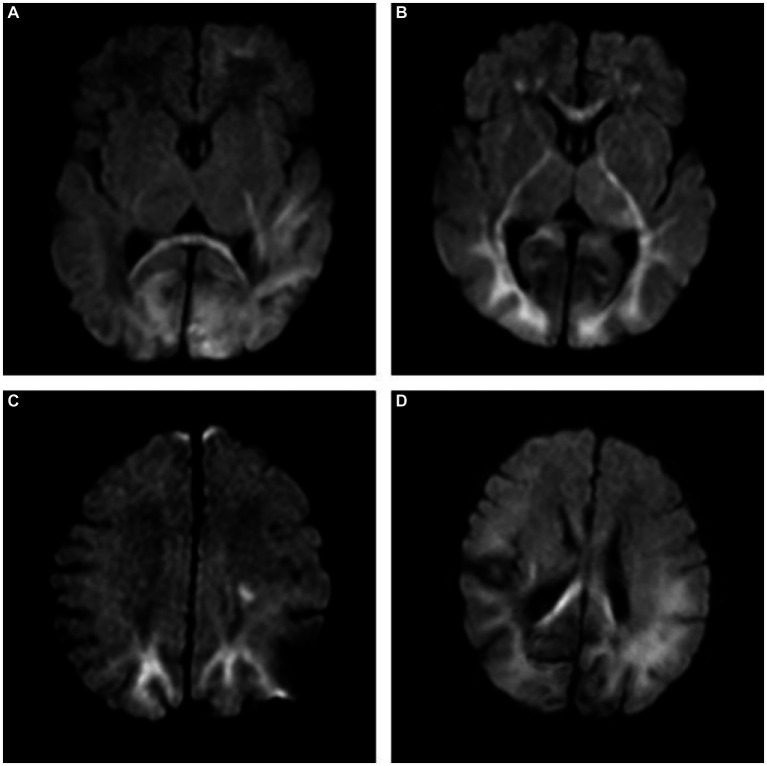
**(A)** A 7-day-old female neonate with HE, panel **(A)** diffusion-weighted imaging (DWI) revealed hyperintense lesions in the bilateral occipital lobe, the left frontal lobe, temporal lobe and the splenium of the corpus callosum, with the outcome of severe neurodevelopmental impairment, included in group B. **(B,C)** A 10-day-old male neonate with HE, panel **(B)** DWI revealed hyperintense lesions in the white matter of bilateral frontal lobe, occipital lobe, internal capsule, thalamus, and genu of the corpus callosum. **(C)** DWI revealed hyperintense lesions in the bilateral parietal lobe and centrum semiovale. **(D)** A 5-day-old female neonate with HE: DWI showed extensive lesions in the brain, included in group B.

**Figure 6:**
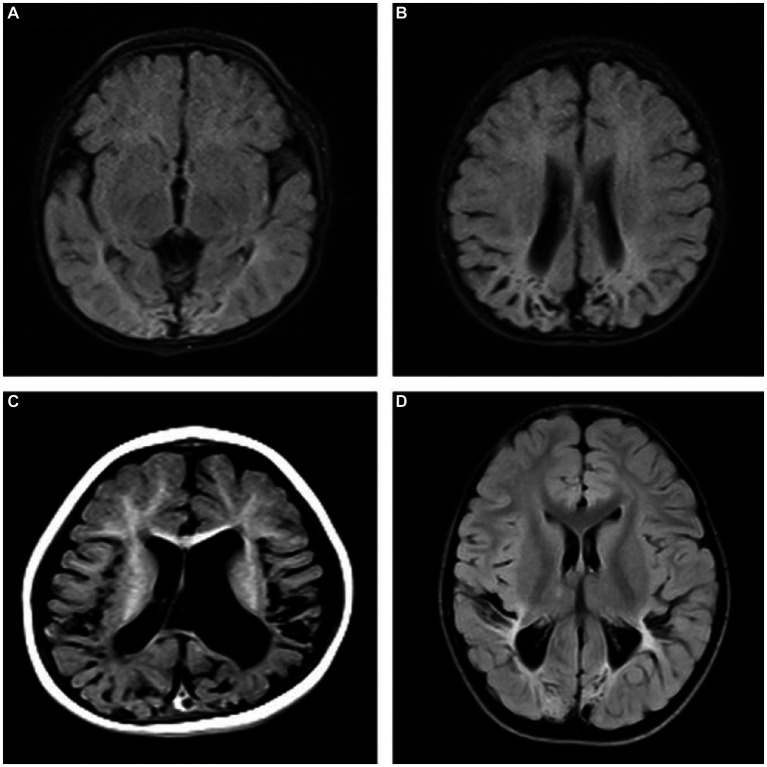
Follow-up outcomes. **(A,B)** A 10-month-old female patient in group A, FLAIR showed bilateral occipital and parietal atrophy, encephalomalacia, ventriculomegaly. **(C)** A 9-month-old male patient in group B: T1W showed bilateral temporal and parietal atrophy, encephalomalacia, ventriculomegaly, delayed myelination and brain sulci widened. **(D)** A 12-month-old male patient in group B, FLAIR showed bilateral occipital and parietal atrophy, encephalomalacia, decreased periventricular white matter, delayed myelination.

The intra- and inter-observer agreement between the two radiologists was acceptable for all parameters (the ICCs ranged from 0.904–0.946). Quantitative values for various brain lesions obtained by quantitative mapping are shown in Figures 2, 3. We discovered that the T1 and T2 values of the parietal lobe, occipital lobe, center semiovale, periventricular white matter, thalamus, and corpus callosum were higher in group B than in group A (*p* < 0.05). The PD values of the occipital lobe, center semiovale, thalamus, and corpus callosum were higher in group B than in group A (*p* < 0.05). However, there were no significant differences in the T1, T2 and PD values of the frontal lobe, temporal lobe, lentiform nucleus, caudate nucleus and cerebellum between the two groups (*p* > 0.05) (Table 2; Figure 4). DWI showed extensive lesions in the brain (Figure 5), and follow-up outcomes are shown in Figure 6.

**Table 2:** Comparison of T1, T2, and PD in each brain region of HE in the two groups.

	Group A (*n* = 40)	Group B (*n* = 35)	*t*/*z*	*P*
Frontal lobe				
T1 (ms)	1968 ± 228	2334 ± 108	3.54	0.170
T2 (ms)	123 ± 21	132 ± 14	−4.42	0.080
PD (pu)	79 ± 10	76 ± 9	−1.13	0.130
Parietal lobe				
T1 (ms)	1986 ± 178	2232 ± 130	−3.01	0.004*
T2 (ms)	127 ± 19	140 ± 21	−2.77	0.023*
PD (pu)	88 ± 23	87 ± 11	−2.02	0.081
Occipital lobe				
T1 (ms)	1912 ± 206	2418 ± 119	−2.25	<0.001*
T2 (ms)	110 ± 15	139 ± 18	−2.85	<0.001*
PD (pu)	96 ± 12	117 ± 14	−3.11	0.029*
Temporal lobe				
T1 (ms)	2084 ± 175	2109 ± 212	−2.10	0.309
T2 (ms)	122 ± 11	114 ± 19	−2.95	0.124
PD (pu)	88[70,108]	86[61,102]	−3.84^#^	0.212
Centrum semiovale				
T1 (ms)	1941 ± 184	2120 ± 223	−3.12	0.002*
T2 (ms)	112 ± 22	127 ± 11	−4.16	0.003*
PD (pu)	90 ± 12	115 ± 19	−3.13	0.006*
Periventricular white matter				
T1 (ms)	2036 ± 103	2318 ± 162	−4.13	0.006*
T2 (ms)	130 ± 21	147 ± 10	−0.98	0.030*
PD (pu)	92 ± 13	84 ± 21	2.97	0.113
Thalamus				
T1 (ms)	1341 ± 106	1581 ± 113	−2.95	0.003*
T2 (ms)	101 ± 10	119 ± 13	2.29	0.010*
PD (pu)	84 ± 17	107 ± 11	−3.24	0.004*
Lentiform nucleus				
T1 (ms)	1499 ± 127	1504 ± 103	−3.14	0.060
T2 (ms)	79 ± 14	64 ± 15	−4.78	0.098
PD (pu)	82 ± 20	96 ± 10	2.21	0.330
Caudate nucleus				
T1 (ms)	1532 ± 211	1603 ± 102	−2.63	0.112
T2 (ms)	124[69,142]	108[80,137]	−2.01^#^	0.070
PD (pu)	87 ± 19	92 ± 13	−3.20	0.085
Corpus callosum				
T1 (ms)	1624 ± 87	1876 ± 24	−2.75	0.020*
T2 (ms)	94 ± 20	113 ± 10	−3.66	0.021*
PD (pu)	82 ± 18	105 ± 21	−2.08	0.005*
Cerebellum				
T1 (ms)	1704 ± 164	1633 ± 108	2.43	0.153
T2 (ms)	133 ± 29	129 ± 15	3.20	0.096
PD (pu)	85 ± 12	94 ± 13	1.13	0.232

^#^Skewed distribution, z; *Significant difference.

### Diagnostic performance

Multivariate logistic regression analysis showed that the duration of hypoglycemia, NBNA score, T1 and T2 value of the occipital lobe, T1 value of the corpus callosum, and T1 value of the thalamus were significant predictors of poor prognosis of HE. The longer the duration of hypoglycemia and the lower the NBNA scores, the worse the prognosis was. The higher T1 and T2 values in the occipital lobe and the higher the T1 values in the corpus callosum and thalamus were, the worse the prognosis was (*p* < 0.05), as shown in Table 3.

**Table 3:** Multivariate logistic regression analysis of statistically significant parameters.

Parameters	*SE*	*Wald*	*OR*	*P*	95% *CI*	
Duration of hypoglycemia	0.013	1.875	2.433	0.002	1.183	~3.098
NBNA score	0.006	0.659	3.094	0.017	1.006	~4.319
T1 value of occipital lobe	0.006	1.052	2.109	0.001	1.611	~5.941
T2 value of occipital lobe	0.012	0.348	2.547	0.001	1.034	~3.941
T1 value of thalamus	0.049	1.165	1.196	0.002	0.879	~3.041
T1 value of corpus callosum	0.014	0.611	1.359	0.003	0.810	~2.606

NBNA, neonatal behavioral neurological assessment; SE, standard error; OR, odds ratio; CI, confidence interval.

**Figure 7:**
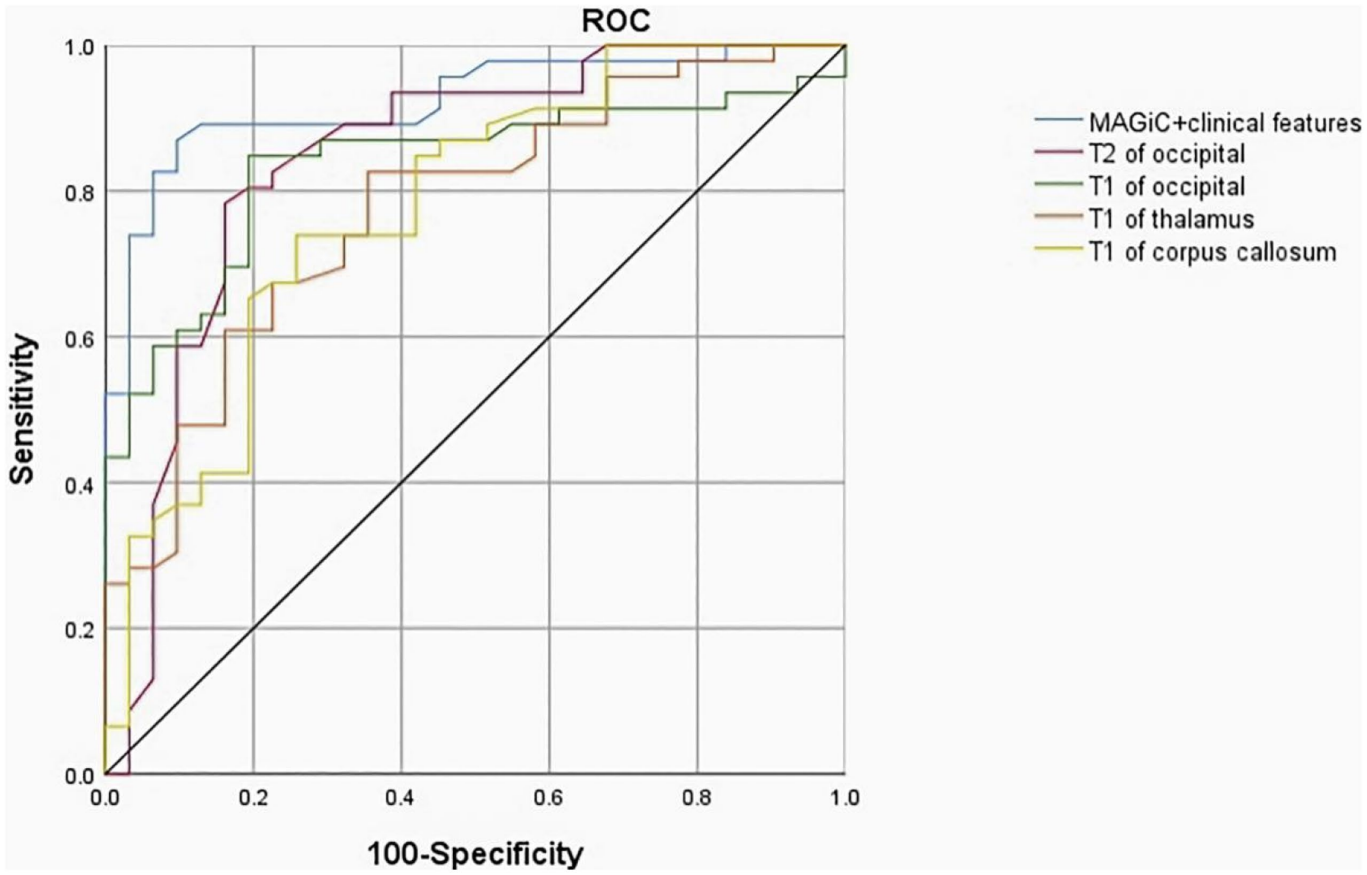
The ROC curve indicated that MAGiC+clinical features have the greatest diagnostic efficacy for poor prognosis of HE (AUC = 0.923, sensitivity 89.12%, specificity 90.63%), followed by T2 value of the occipital lobe (AUC = 0.844, sensitivity 83.02%, specificity 88.16%). *ROC*, receiver operating characteristic; *AUC*, area under the curve.

ROC analysis showed that the AUC values of MAGiC quantitative values predicting the prognosis of HE ranged from 0.719–0.844. The T2 values of the occipital lobe showed the best diagnostic performance among all MAGiC-derived single parameters for predicting prognosis, with an AUC value of 0.844, sensitivity of 83.02%, and specificity of 88.16%. Furthermore, the combination of MAGiC quantitative values and perinatal clinical features (duration of hypoglycemia and NBNA score) can improve the AUC compared with MAGiC or perinatal clinical features alone, with an AUC value of 0.923, sensitivity of 89.12%, and specificity of 90.63%. The *p* value of DeLong test was 0.014, as shown in Figure 7 and Table 4.

**Table 4:** Diagnostic performance of quantitative MAGiC.

Parameters	*AUC*	*Cutoff*	*YI*	*Sen*(%)	*Spe*(%)	*P*	95% *CI*	
MAGiC+clinical features	0.923	0.697	0.796	89.12	90.63	<0.001	0.845	~1.000
T1 value of occipital lobe	0.835	2312.2	0.528	82.65	70.10	0.001	0.772	~0.914
T2 value of occipital lobe	0.844	132.5	0.712	83.02	88.16	<0.001	0.687	~0.950
T1 value of thalamus	0.774	1516.5	0.562	75.14	81.05	0.002	0.652	~0.870
T1 value of corpus callosum	0.737	1725.8	0.543	78.09	76.34	0.003	0.683	~0.781

*YI*, Youden index; *Sen*, sensitivity; *Spe*, specificity; *CI*, confidence interval.

## Discussion

Neonatal hypoglycemia causes great harm, especially long-term or recurrent hypoglycemia, which can cause severe brain function injury, resulting in delayed brain development, intellectual disability and even death in children. Therefore, it is very important to screen out early prognostic risk factors by combining imaging and clinical characteristics (Landais, 2015; Kapoor et al., 2020; Van Kempen et al., 2020; Parmentier et al., 2022). Mckinlay et al. found that in a 15-month neurodevelopmental follow-up, the intelligence and motor development indices of children with hypoglycemia accompanied by convulsions were significantly lower than those of children with mild symptoms or asymptomatic hypoglycemia (Mckinlay et al., 2017), and studies have detected that asymptomatic hypoglycemia can also cause neurological sequela (Hassan et al., 2020; Zhang et al., 2022). These studies suggested that children with hypoglycemia accompanied by symptoms or severe symptoms for a long time are prone to neurological sequela.

In our study, there were statistically significant differences in birth weight, history of asphyxia, history of gestational diabetes or gestational hypertension, duration of hypoglycemia and NBNA scores between the two groups, while duration of hypoglycemia and NBNA scores were associated with poor prognosis. It is proposed that for individuals with low body weight, history of asphyxia, maternal pregnancy-induced hypertension and other risk factors, neonatal hypoglycemia should be screened timely and monitored continuously (Mckinlay et al., 2017). The NBNA is one of the main means to investigate the neurological development of newborns, and it was developed by Chinese neonatologist Bao based on the Amiel-Tison neuromotor measurement method from France and the Brazelton neonatal behavior score from the United States (Bao et al., 1993). It can comprehensively evaluate neonatal behavioral ability and various nerve reflexes. Moreover, it is more accurate and complete than traditional neurological tests. It is simple to operate, has high sensitivity and specificity values, and can be used as a reliable index for early assessment. A strong relationship between NBNA scores and brain development outcome has been reported (Nin et al., 2011). Our study showed that the lower the NBNA score is, the worse the prognosis of neonatal HE, which was consistent with previous studies (Kim et al., 2006; Wang et al., 2014; Hori et al., 2020). Attention should be given to NBNA in the perinatal period, and early intervention should be conducted for those with abnormal scores.

The difference in relaxation time across different tissues in MRI is related to free water content in tissues, random movement of water molecules and macromolecules, tissue fat content and the presence or absence of a paramagnetic substance (Nelson et al., 2007; Granberg et al., 2016), among which free water content has the most obvious influence. T1 can reflect the change in free water content, myelination degree and iron content in the brain, and the decrease in the myelin sheath and the increase in free water in the brain are two of the reasons for the increase in T1 values. T2 is considered to be a reliable and repeatable biomarker of flow fluid content in different tissues. The lower the water content in the tumor tissue, the lower the T2 value is (Spieker et al., 2018; Zhao et al., 2021). Moreover, backward myelination has been shown to be a possible cause of high T2 values in the white matter (Moritani et al., 2008). PD, as another magnetic property of tissue, reflects the difference in hydrogen proton content in different tissues, and its increase also indicates the increase in water content in the measured area. A previous syMRI study found that PD could distinguish benign from malignant metastatic retropharyngeal lymph nodes (Wang et al., 2023). Quantitative MAGiC maps are effective in showing T1, T2, and PD, reflecting the myelination process in the affected area (Gracien et al., 2016; Andica et al., 2019; Di Giuliano et al., 2020), and they are not affected by scanning time. The metabolism of the neonatal brain is vigorous, the demand for glucose is great, and the decrease in blood glucose levels makes the energy supply of the brain insufficient. Due to the lack of ATP and phosphocreatine, the opening of calcium channels in the cell membrane causes cytotoxic edema, and water molecules enter the cell, eventually leading to the degeneration and necrosis of nerve cells, increased vascular permeability and glial cell proliferation. Therefore, the more severe the lesions in the HE, the higher the T1, T2 and PD values were. The bilateral parietal and occipital lobes are the most vulnerable regions, and the corpus callosum is also a vulnerable area. However, the patterns of brain injury are complex and varied (Sharp and DeMaruo, 2017), usually involving multiple sites. Burns et al. suggested that brain injury in neonatal HE is not only confined to the parietal and occipital lobes but also involve the deep gray matter and white matter (Burns et al., 2008). A clinical study found that the splenium of the corpus callosum is a vulnerable region for HE in adults (Righini et al., 2010). This exploratory study showed that the values of T1 and T2 in the parietal lobe, occipital lobe, centrum semiovaler, periventricular white matter, thalamus and corpus callosum in HE were higher in the severe group than in the normal and mild group; additionally, PD values in the occipital lobe, centrum semiovaler, thalamus, and corpus callosum in HE were significantly increased with the severity of the disease. Our results were consistent with the affected parts in previous studies (Wang et al., 2014). This may be because the primitive visual cortex in the parietal lobe and occipital lobe is larger and includes more neurons and synapses; therefore, it is more sensitive to hypoglycemia (Kim et al., 2006; Mckinlay et al., 2017; Van Kempen et al., 2020). Hypoglycemia leads to increased free water and blocked myelination. In addition, we discovered that the differences in T1, T2 and PD in the thalamus between the two groups were statistically significant. In previous studies, thalamus involvement in HE was less common. Misser SK et al. noted that the risk of a thalamus L-sign injury was 2.79 times higher when both the parietal and occipital lobes were injured compared with when they were not involved in HE (Misser et al., 2022). The thalamus is a central hub that connects several brain structures as well as the cerebellum and spinal cord. Damage to the thalamus provides an indirect index of tract-based injuries to the rest of the brain, and we can infer damage patterns that can be attributed to a specific pathophysiologic process (Burns et al., 2008; Squarcina et al., 2012).

However, there were no significant differences in T1, T2 and PD values in the frontal lobe, temporal lobe, lentiform nucleus, caudate nucleus and cerebellum between the two groups, which may be due to the fact that the neuronal structure of these parts is less sensitive to glucose than that of the parietal lobe, occipital lobe and corpus callosum during the neonatal period. Logistic regression analysis showed that T1 and T2 values in the occipital lobe and T1 values in the corpus callosum and thalamus were risk factors for poor prognosis. The observation of these parameters was focused on providing quantitative and reproducible results for the prognosis of neonatal HE in the early stage. ROC analysis showed that quantitative analysis of MAGiC predicted the prognosis of HE with an AUC ranging from 0.719 to 0.844, among which the AUC of T2 in the occipital lobe was the largest (AUC = 0.844). Combined with the duration of hypoglycemia, NBNA scores and quantitative analysis in MAGiC, the AUC increased to 0.923, which indicated that quantitative analysis in MAGiC combined with clinical characteristics can predict the prognosis of neonatal HE more effectively.

Quantitative analysis of MAGiC adopted in this study displayed high reproducibility, multiple diagnostic parameters, and high diagnostic efficiency. MAGiC is a novel technique to evaluate neonatal HE quantitatively, which played a crucial role in the prognosis evaluation and provided a basis for treatment. Despite the great potential, considerable challenges remain, such as standardization, reproducibility, and accuracy. These issues should be addressed before they can play a role in clinical decision-making. Additional follow-up is needed to verify the accuracy of the study. However, MAGiC has limitations. Compared with conventional FLAIR images, synthetic FLAIR images have inferior image quality (lower contrast-to-noise ratio) (West et al., 2017; Fujioka et al., 2021). Furthermore, due to partial volume effects, the interface between the brain parenchyma and cerebrospinal fluid (CSF) is hyperintense on a synthetic FLAIR image; therefore, a conventional FLAIR image needs to be obtained. However, errors could be avoided if the images are observed carefully.

We suggest that the quantitative value of MAGiC may better reflect pathophysiological changes caused by HE, but further studies are essential to test this hypothesis. We believe that quantitative mapping of images generated by MAGiC can be used to measure of the severity of disease and to predict prognosis.

Our work has some limitations. (1) Slight errors occur when ROI is manually delineated, thus affecting the accuracy of the data. (2) The late onset of hypoglycemia in some children and the delay of neurological symptoms may lead to the delay of MR examination, and some lesions are in the stage of false normalization (Thompson-Branch and Havranek, 2017; Comtié de Estudios Feto-Neonatales, 2019), which may interfere with the results to a certain extent. (3) The ROIs we selected were all in white matter. There will be a large delineation error in consideration that the gray matter of newborns is thin. We ignored the quantitative analysis of gray matter, which needs added improvement. (4) The sample size obtained in this study was small, and it is necessary to examine larger cohorts to determine the range of T1, T2 and PD values of different severities in neonatal HE. (5) Although patients with hypoxic–ischemic encephalopathy were excluded from the study, gestational hypertension or gestational diabetes may also cause brain injury caused by asphyxia, which overlaps with HE. Therefore, we should improve the deficiencies in the future to provide more valuable evidence for clinical practice.

In conclusion, MAGiC can be used to obtain the corresponding T1 and T2 relaxation times and PD quantitative map by various combinations of TR, TE and TI, which can eliminate other signals and reflect the differences in the T1 and T2 relaxation times and hydrogen proton content in different tissues, respectively. Quantitative analysis of syMRI has better prognostic value for neonatal HE, and the prognostic efficiency combined with clinical characteristics is further optimized.

## Data availability statement

The raw data supporting the conclusions of this article will be made available by the authors, without undue reservation.

## Ethics statement

The studies involving human participants were reviewed and approved by Medical Ethics Committee, Women’s Hospital of Nanjing Medical University (Nanjing Maternity and Child Health Care Hospital). Written informed consent to participate in this study was provided by the participants’ legal guardian/next of kin.

## Author contributions

ZT and RW collected the data and drafted the manuscript. YX and QZ analyzed the data. WT and MY guided and revised the manuscript throughout. All authors contributed to manuscript development, and approved the final manuscript.

## Funding

This study was supported by the Nanjing Science and Technology Development Fund, CN(YKK14117).

## Conflict of interest

The authors declare that the research was conducted in the absence of any commercial or financial relationships that could be construed as a potential conflict of interest.

## Publisher’s note

All claims expressed in this article are solely those of the authors and do not necessarily represent those of their affiliated organizations, or those of the publisher, the editors and the reviewers. Any product that may be evaluated in this article, or claim that may be made by its manufacturer, is not guaranteed or endorsed by the publisher.
